# Inclusion of different levels of fermented elephant grass in broiler chicken diet: effects on growth, physiology, carcass traits and gut microbial community

**DOI:** 10.3389/fphys.2026.1767570

**Published:** 2026-03-20

**Authors:** Samaila Usman, Qi Yan, Lijuan Gao, Suyuan Deng, Liyan Lu, Tiande Pang, Dawei Lai, Chris S. Jones, Xianfeng Yi, Jiyu Zhang

**Affiliations:** 1 State Key Laboratory of Herbage Improvement and Grassland Agro-Ecosystems, College of Pastoral Agriculture Science and Technology, Lanzhou University, Lanzhou, China; 2 School of Life Sciences, Lanzhou University, Lanzhou, China; 3 Guangxi Vocational University of Agriculture, Nanning, China; 4 Feed and Forage Development, International Livestock Research Institute, Nairobi, Kenya

**Keywords:** elephant grass, broiler performance, carcass traits, blood biochemistry, gut microbiota

## Abstract

The physiological and microbiome-modulating benefits of dietary forage in monogastrics are impeded by recalcitrant fiber and anti-nutritional factors. However, fermentation and appropriate inclusion levels may overcome these limitations. This study evaluated the effects of two fermented cultivars of elephant grass (*Cenchrus purpureus* cv. Guiminyin and cv. Purple) incorporated into broiler diets at different inclusion levels, with emphasis on cultivar-specific responses, growth performance, physiological status, and gut microbial composition. A total of 240 male (30-days old) Jinling earth-neck chickens were housed in four replicate pens of 12 birds each, and randomly assigned to five dietary treatments (48 birds per treatment): a control diet with no inclusion (0%), CpGui5 (5% Guiminyin inclusion), CpGui10 (10% Guiminyin inclusion), CpPur5 (5% Purple inclusion), and CpPur10 (10% Purple inclusion). CpGui5 and Control diets had statistically similar and higher final weight, total weight gain, average daily gain and feed efficiency which were higher than the other treatments (*P* < 0.05)*.* On the other hand, Control, CpGui5 and CpPur5 had higher daily feed intake compared to CpGui10 and CpPur10 which had the lower daily feed intake (*P* < 0.05). Slaughter performance revealed significant differences (*P* < 0.05), with the control, CpGui5, CpPur5 and CpPur10 groups maintaining higher live weight, dressed weight, half-bore, and breast muscle rate while CpGui10 recorded the lowest values. Blood biochemical indices, including total protein, albumin, liver enzymes, and renal function markers, were unaffected by dietary treatments (*P* > 0.05), indicating no adverse physiological effects. Gut microbiome analysis showed stable richness (Chao1, ACE) across treatments, while diversity (Shannon, Simpson) was reduced in CpPur5 relative to other groups (*P* < 0.05). A shared core microbiome of 202 OTUs was detected across all treatments, alongside treatment-specific enrichment of taxa. LEfSe (Linear Discriminant Analysis Effect Size) analysis identified treatment-specific enrichment of functionally relevant bacterial genera, including *Megamonas* in CpGui5 and *Ruminococcaceae_UCG-014* and unclassified *Lachnospiraceae* at higher inclusion levels (CpGui10). Overall, moderate inclusion of fermented elephant grass, especially CpGui5 supports broiler performance while maintaining physiological health and gut microbial stability, highlighting its potential as a sustainable functional feed resource for poultry production.

## 1 Introduction

Global poultry production intensification faces critical sustainability challenges stemming from heavy reliance on conventional feed resources such as maize and soybean, which drive land-use competition, food-feed conflicts, and supply chain vulnerabilities (Pexas et al., 2023). These challenges have stimulated interest in alternative feed resources capable of partially substituting conventional ingredients while maintaining growth performance and physiological stability in broilers. Hence, the need for exploring alternative feed from unconventional resources such as forages. Tropical forages such as *Cenchrus purpureus* (Schumach) (elephant grass) provide a rich biomass that can serve as an alternative feed due to their high yield potential and adaptability to marginal lands (Islam et al., 2023). Depending on the cutting age, elephant grass contains crude protein and fiber levels ranging between 12.70%–6.53% and 48.79%–72.99%, respectively (Botero-Londoño et al., 2021). Nevertheless, its utilization in broiler nutrition remains constrained by the high fiber content, lignin complexity, and associated anti-nutritional factors that limit nutrient bioavailability in monogastrics. Hence, appropriate processing strategies such as fermentation are required to enhance its nutritional suitability for poultry feeding systems.

Microbial fermentation is an established bioprocessing approach for improving the feeding value of fibrous plant materials. During fermentation, microbial enzymatic activity partially deconstructs complex cell wall polysaccharides, reduces anti-nutritional factors, and generates organic acids and other metabolites that can enhance feed stability and digestibility (Du et al., 2024). These transformations may also influence the gastrointestinal environment by altering substrate availability for intestinal microbes and modifying gut physicochemical conditions (Huang et al., 2023). The application of fermented feedstuffs in poultry production remains nascent, with critical knowledge gaps persisting. It offers a cost-effective method to enhance unconventional broiler feed by improving gut health, immunity, and growth while reducing pathogen colonization, offering potential for lower feed costs and sustainable production (Sugiharto and Ranjitkar, 2019). Diet-induced changes in the gut microbiota are increasingly recognized as important mediators linking feed composition to host metabolism, immune function, and growth performance (Ogbuewu et al., 2024). However, responses to fermented feed ingredients are highly dependent on substrate characteristics, processing conditions, inclusion level, and bird developmental stage, underscoring the need for systematic evaluation (Katu et al., 2025).

Elephant grass cultivars differ especially in bioactive compound profiles due to pigmentation, a factor that may influence fermentation dynamics biological responses in broilers. However, limited information is available on the dose-dependent effects of fermented elephant grass from different cultivars when incorporated into broiler diets. Potential trade-offs between growth performance, carcass traits, meat quality attributes, and physiological indicators have not been comprehensively assessed. The extent to which fermented elephant grass modulates the gut microbial community as a mechanistic contributor to these outcomes also remains poorly understood.

Therefore, the present study evaluated the effects of fermented elephant grass incorporated into broiler diets at graded inclusion levels, with emphasis on cultivar-specific responses. Using two elephant grass cultivars (Guiminyin and Purple), we assessed growth performance, carcass characteristics, physiological and biochemical indices, and ileal microbial community structure. This study provides evidence-based insight into the feasibility of using fermented forages as alternative feed ingredients in broiler production and contributes to a clearer understanding of diet–microbiome–host interactions in poultry nutrition.

## 2 Materials and methods

### 2.1 Diet preparation

*Cenchrus purpureus* Schumach cv. Guiminyin (CpGui) and cv. Purple (CpPur) were vegetatively propagated at the Guangxi Institute of Animal Sciences, Guangxi Province, China, in March 2017. Plants were grown under rainfed conditions, with nitrogen and compound fertilizers applied every 40 days. At the time of cutback for this study, the crops were 1.5 years old. Fresh elephant grass forage with 80 days of regrowth was cut 10 cm above ground level and crushed to 1 cm length using a crop straw rubbing filament machine (9RC-50, Zhengzhou Muchang Agricultural Machinery Manufacturing Co., Ltd.). The dry matter (DM) content of the crushed grasses was adjusted to approximately 45%–55%. Subsequently, 10% dried corn flour was added to each cultivar (on dry matter basis) along with 0.5% micro-stock feed fermenting agent (a compound probiotic containing: *Lactococcus lactis, Saccharomyces cerevisiae,* cellulase and xylanase), which was sprayed evenly onto each cultivar. The treated grasses were compacted using a punch press, sealed in polyethylene bags, and fermented anaerobically for 30 days. After fermentation, the chemical compositions of the fermented grasses were similar (17.5% crude protein, 2.46% crude fat, 20.4% crude fiber, 8.87% ash, 66.1% neutral detergent fiber, 41.1% acidic detergent fiber, 0.40% calcium, and 0.46%. phosphorus), and they were subsequently used for experimental diet formulation. The experimental dietary treatments include a control diet, the control diet supplemented with 5% fermented Guiminyin cultivar (CpGui5) or 10% (CpGui10), and the control diet supplemented with 5% fermented Purple cultivar (CpPur5), or 10% (CpPur10). The composition and nutrient levels of the control and the fermented elephant grass-incorporated diets are presented in Table 1.

**TABLE 1:** Ingredients and chemical composition of experimental diets.

Ingredient composition	Treatments				
	Control	CpGui5	CpGui10	CpPur5	CpPur10
Wheat bran (%)	5.03	4.49	4.02	4.49	4.02
Compound premix (%)	4.02	3.62	3.22	3.62	3.22
Fermented elephant grass (%)	0	5.0	10	5.0	10
Maize (%)	71.36	71.49	63.72	71.49	63.72
Soybean meal (%)	19.59	15.4	19.04	15.4	19.04
Nutrient levels					
Crude fibre (% DM)	3.08	3.15	3.28	3.98	4.61
Crude protein (% DM)	17.2	18.6	18.6	19.4	20.9
Metabolizable energy (Mcal/kg)	3.13	3.28	3.28	3.29	3.31

### 2.2 Chicken’s management and experimental design

Before the experiment, the research facilities were thoroughly cleaned and disinfected. A total of 240 30-days-old male chickens of the Jinling earth neck breed, purchased from Guangxi Jinling Agriculture and Animal Husbandry Co., Ltd. (Nanning, China). The chickens were housed in four replicate pens of 12 birds each (48 birds per treatment) with each pen measuring 1.5 × 1.0 m. Each of the four replicate groups were randomly assigned to the five treatments diet shown in Table 1. Temperature in the chicken house was maintained at 24 °C with constant lighting of 24 h throughout the feeding trial. Feed was provided *ad libitum* and the birds had free access to water throughout the entire experimental period which lasted for 71 days. All the experimental protocols were approved by the Animal Ethics Committees of Guangxi Institute of Animal Sciences (file NO: 2017-1 and 2017-2).

### 2.3 Growth performance and carcass characteristics

Daily feed intake (DFI) was recorded every day, while body weight (BW) of the was recorded weekly. These parameters were used to calculate average daily gain (ADG), total weight gain (TWG) and feed efficiency (FE). At the age of 101 days, after 4 h of fasting, four chickens per treatment (one from each replicate) that had weights closest to the mean weight for the cage were selected, and then weighed and exsanguinated by cutting their jugular vein. Carcasses were then defeathered and eviscerated to determine carcass weight (without head paws or giblets) as a percentage of total weight. The abdominal adipose tissue (from the proventriculus surrounding the gizzard down to the cloaca), breast muscle, and leg muscle from each bird were collected and weighed. Carcass dressing percentage, abdominal fat, breast muscle, and leg muscle were expressed as a percentage of BW.

### 2.4 Measurement of blood parameters

Blood samples were collected at slaughter. Birds were humanely euthanized in accordance with institutional animal care and guidelines. During euthanasia, the blood was collected from carotid artery before complete severing, and out directly into sterile collection tubes. The samples were placed in an icebox and transported promptly to the laboratory for the analysis of blood constituents. After centrifuging blood samples (3,000 x g, for 20 min at room temperature), plasma was harvested and stored in Eppendorf tubes at −20 °C until assayed. The concentrations of serum total protein (TP), albumin (ALB), globulin (GLO) and blood urea nitrogen (BUN) were determined using commercial kits (Shanghai Kehua bioengineering Co., Ltd., Shanghai, China) while albumin to globulin ratio (A/G) was estimated as ratio of ALB to GLO. Aspartate aminotransferase (AST), alanine transaminase (ALT), alkaline phosphatase (ALP), total bile acids (TBA), cholinesterase (CHE), creatinine (CRE-E), uric acid (UA).and γ-glutamyltransferase (γ-GT) in the plasma were measured using an Automatic Biochemical Analyzer (Shenyang Pulide Trading Co., Ltd., China).

### 2.5 16S rDNA sequencing and analysis

Gut digesta samples were collected from the cecal contents of four birds per treatment immediately after slaughter. Both ceca were aseptically excised, opened longitudinally using sterile scissors, and the luminal contents were gently collected into sterile, DNA-free microcentrifuge tubes. Samples were placed on ice during collection and subsequently stored at −80 °C until DNA extraction. The collected material was used directly for downstream DNA extraction using standard commercial microbial DNA extraction kits (TianGen Biotech Co. Ltd., Beijing, China).

DNA concentration was quantified using a Qubit 2.0 Fluorometer (Invitrogen, Carlsbad, CA, United States of America). Amplicon libraries were prepared using the MetaVx™ Library Preparation Kit (GENEWIZ, South Plainfield, NJ, United States of America) with 30–50 ng DNA per sample. The V3–V4 regions of the prokaryotic 16S rDNA were amplified using GENEWIZ-designed primers targeting conserved regions flanking the V3–V4 hypervariable regions (forward: CCTACGGRRBGCASCAGKVRVGAAT; reverse: GGACTACNVGGGTWTCTAATCC). First-round PCR products were used for a second-round enrichment PCR in which indexed adapters were added. Final libraries were validated using an Agilent 2100 Bioanalyzer, quantified with Qubit 2.0, pooled, and sequenced on an Illumina MiSeq platform (Illumina, San Diego, CA, United States of America) using a 2 × 300 bp paired-end configuration.

Raw sequences were processed in QIIME. Paired-end reads were merged, demultiplexed, and trimmed to remove primers and barcodes. Quality filtering removed reads <20 bp. Chimeras were identified using UCHIME against the RDP Gold database and discarded. High-quality reads were clustered into OTUs at 97% similarity using VSEARCH (v1.9.6) against the SILVA 119 database. Taxonomy of representative OTU sequences was assigned with the RDP classifier (confidence threshold 0.8) using the SILVA 123 database. Sequences were rarefied prior to diversity analyses. Alpha-diversity indices were calculated to assess species’ richness and evenness, and beta-diversity metrics were used to evaluate differences in community composition among samples.

### 2.6 Statistical analysis

Growth performance, blood profile, and carcass characteristics data were subjected to one-way ANOVA using RStudio of Posit Software (R Core Team, 2024). Significant means were separated using Tukey’s’ HSD and significance was declared when the probability was less than 5% (*P* < 0.05). The alpha and beta diversity were also calculated in RStudio using Microeco package (Liu et al., 2021) while figures were generated using ggplot2 package (Wickham, 2016).

## 3 Results

### 3.1 Growth performance

Growth performance of chickens fed control diet and diets containing fermented elephant grass including the initial and final weight, TWG, DFI, ADG and FE are presented in Table 2. The table shows that chickens fed CpGui5 and Control diets had statistically similar final weight, TWG, ADG and FE (*P* > 0.05). However, the CpGui5 and Control had significantly higher values for the mentioned growth parameters and FE (*P* < 0.05) compared to the remaining treatments (CpPur5, CpGui10 and CpPur10). On the other hand, Control, CpGui5 and CpPur5 had higher DFI compared to CpGui10 and CpPur10 which had the lower DFI (*P* < 0.05). Notably, the control treatment also had statistically similar TWG, ADG and FE with CpPur5 treatment FE (*P* > 0.05).

**TABLE 2:** Performance of chickens fed control diet and diets containing fermented elephant grass.

Item	Treatments					SE	*P*-values
	Control	CpGui5	CpGui10	CpPur5	CpPur10		
Initial weight, kg	0.84	0.84	0.79	0.82	0.80	0.012	0.074
Final weight, kg	1.89^a^	1.91^a^	1.71^a^	1.77^a^	1.71^a^	0.033	0.001
TWG, kg	1.06^a^	1.08^a^	0.91^a^	0.95^a^	0.90^a^	0.025	<0.001
DFI, g/day	94.25^a^	94.25^a^	91.77^a^	94.25^a^	91.77^a^	0.000	<0.001
ADG g/day	15.10^a^	15.40^a^	13.05^a^	13.54^a^	12.91^a^	0.358	<0.001
FE	0.160^a^	0.163^a^	0.142^a^	0.144^a^	0.141^a^	0.004	0.001

^a^ Means in the same row with different superscripts are significantly different (*P* < 0.05).

TWG, total weight gain; DFI, daily feed intake; ADG, average daily gain; FE, feed efficiency.

### 3.2 Slaughter performance and carcass traits

Table 3 shows that the live weight, dressed weight, half-bore and breast muscle rate were significantly different among the chickens fed control diet and diets containing fermented elephant grass (*P* < 0.05). The control treatment which has statistically similar value with CpGui5, CpPur5 and CpPur10 recorded higher values for the live weight, dressed weight, and half-bore than CpGui10 which recorded the lowest values of 1.69 kg, 1.56 kg, and 1.25, respectively (*P* < 0.05). The breast muscle rate was higher in control, CpGui5, and CpPur5 compared to CpGui10 and CpPur10 (*P* < 0.05). However, full bore, abdominal weight, leg muscle rate and spleen weight differences were not significant among the elephant grass incorporated treatments and the control (*P* > 0.05*).*

**TABLE 3:** Slaughter performance and carcass traits of chickens fed control diet and diets containing fermented elephant grass.

Item	Treatments					SE	*P-*values
	Control	CpGui5	CpGui10	CpPur5	CpPur10		
Live weight, kg	2.00^a^	1.91^a^	1.69^a^	1.81^a^	1.85^a^	0.052	0.001
Dressed weight, kg	1.86^a^	1.77^a^	1.56^a^	1.69^a^	1.69^a^	0.053	0.005
Half-bore	1.57^a^	1.53^a^	1.35^a^	1.44^a^	1.48^a^	0.050	0.031
Full bore	1.3	1.2	1.1	1.2	1.2	0.061	0.215
Abdominal fat weight, kg	0.11	0.10	0.07	0.08	0.10	0.012	0.242
Breast muscle rate	0.10^a^	0.09^a^	0.08^a^	0.09^a^	0.09^a^	0.004	0.020
Leg muscle rate	0.13	0.12	0.11	0.12	0.12	0.006	0.279
Spleen weight	1.73	1.95	1.57	1.88	1.61	0.126	0.164

^a^ Means in the same row with different superscripts are significantly different (*P* < 0.05).

### 3.3 Physiological performance and biochemical parameters

The biochemical parameters of the chickens fed control diet and diets containing fermented elephant grass are presented in Table 4. The table shows that all the blood biochemical parameters including TP, ALB, GLO, A/G, ALT, AST, γ-GT, ALP, TBA, CHE, BUN, CRE-E and UA had no significant differences among the elephant grass incorporated treatments and control (*P* > 0.05).

**TABLE 4:** Physiological performance and blood biochemical parameters of chickens fed control diet and diets containing fermented elephant grass.

Item	Treatments					SE	*P*-values
	Control	CpGui5	CpGui10	CpPur5	CpPur10		
TP g/L	41.33	42.67	49.33	50.33	42.33	6.59	0.790
ALB g/L	18.33	19.33	21.00	18.67	16.67	1.92	0.626
GLO g/L	23.00	23.33	28.33	30.00	25.67	4.93	0.813
A/G g/L	0.80	0.84	0.75	0.66	0.78	0.10	0.732
ALT	3.33	5.33	0.67	2.33	2.33	2.68	0.800
AST µ/L	173.33	192.00	174.67	166.33	122.33	17.2	0.131
γ-GT µ/L	23.67	20.67	9.33	24.67	12.00	6.12	0.334
ALP, µ/L	1,411.00	1,408.33	1,634.00	1,442.67	460.67	378	0.274
TBA, µmol/L	20.90	18.47	15.23	9.87	10.20	4.10	0.294
CHE, µ/L	1,532.33	1,512.33	1963.00	1,458.33	1,259.33	241	0.391
BUN, mmol/L	0.20	0.26	0.21	0.25	0.11	0.10	0.805
CRE-E, µmol/L	10.00	10.33	11.67	9.33	8.00	1.41	0.497
UA, µmol/L	231.67	226.33	177.00	231.33	151.67	25.7	0.161

TP: total protein; ALB: albumin; GLB: globulin; A/G: albumin/globulin ratio; ALT: alanine transaminase; AST: aspartate transaminase; γ-GT: gamma glutamyl transferase; ALP: alkaline phosphatase; TBA: total bile acids; CHE: cholinesterase; BUN: blood urea nitrogen; CRE-E: creatinine; UA: uric acid.

### 3.4 Gut microbial community

**FIGURE 1:**
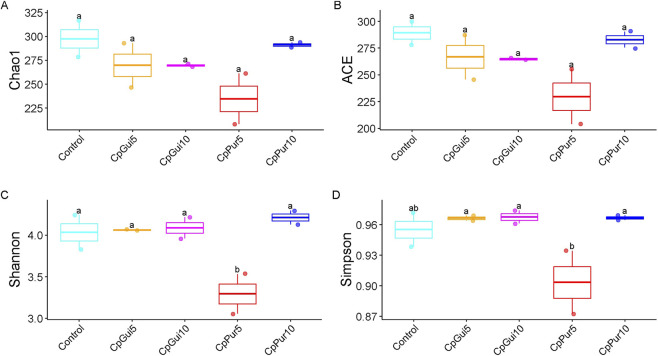
Alpha diversity of gut microbial community of chickens fed control diet and diets containing fermented elephant grass. **(A,B)** Boxes of the same index bearing different letters are significantly different (*P* < 0.05).

The alpha diversity indices including Chao1, ACE Shannon and Simpson of the gut microbial community of chickens fed control diet and diets containing fermented elephant grass are presented in Figure 1. The figure shows that there were no significant differences in Chao1 and ACE among the elephant grass incorporated treatments and the control (*P* > 0.05), although the CpPur5 treatment was consistently the lowest. However, control, CpGui5, CpGui10 and CpPur10 treatments had statistically similar and higher Shannon and Simpson indices than the CpPur5 treatment (*P* < 0.05).

**FIGURE 2:**
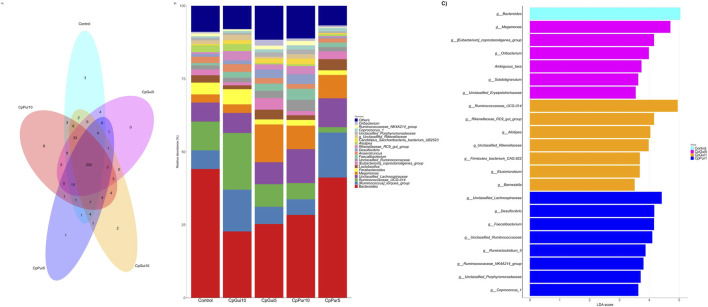
OTU distribution and relative abundance of gut microbial community of chickens fed control diet and diets containing fermented elephant grass. **(A)** Venn diagram showing OTUs distribution among the treatments. **(B)** Genus relative abundance among the treatments. **(C)** Genus linear discriminant analysis (LDA) and effect size measurement (LEfSe).

The Venn diagram illustrates the distribution of bacterial taxa shared and uniquely represented among the different elephant grass incorporated treatments and the control (Figure 2A). A core microbiome of 202 OTUs was consistently detected across all groups, underscoring a stable microbial backbone that persists irrespective of treatment. However, each treatment also harbored distinct microbial populations, indicating treatment-specific modulation of community structure. Notably, CpPur10 exhibited the highest number of unique taxa (8 OTUs), whereas CpGui5 and CpPur5 retained fewer unique members (0 and 1 OTUs, respectively).

The relative abundance of dominant bacterial taxa at the genus level across the different elephant grass incorporated treatments and the control is presented in Figure 2B. The microbial community was dominated by genera such as *Bacteroides* [*Ruminococcus]_torques_group*, *Ruminococcaceae_UCG-014*, *unclassified_Lachnospiraceae, Megamonas*, *Parabacteroides*, and *Lactobacillus*, although their proportions varied among treatments. Different levels of CpGui and CpPur inclusion altered the relative abundance of several dominant taxa compared with the Control. Differences in bar composition indicate diet-driven restructuring of the microbial community, rather than the appearance of entirely new taxa.

Figure 2C presents a LEfSe (Linear Discriminant Analysis Effect Size) identifying bacterial genera that significantly discriminated among treatments (LDA score >3.5). The Control group was characterized by enrichment of *Bacteroides* genera which also has the highest percentage of relative abundance in all treatments. Several genera were identified to have significant abundances and higher LDA scores include *Megamonas* in CpGui5, *Ruminococcaceae_UCG-014* in CpGui10 and *unclassified_Lachnospiraceae* which are among the top five genera with highest parcentage of relative abundance. Notably, there was no distinct genera identified by the LEfSe analysis in the CpPur5 treatment.

## 4 Discussion

Dietary fiber has traditionally raised concerns in poultry nutrition because of its potential to reduce nutrient digestibility and energy utilization. However, increasing evidence indicates that moderate fiber inclusion, particularly from well-processed plant sources, can support gastrointestinal function and feed efficiency without compromising performance (Jha and Berrocoso, 2015; Yadav and Jha, 2019). In the present study, incorporation of fermented elephant grass into broiler diets influenced feed intake and growth performance in a dose- and cultivar-dependent manner. The absence of differences in DFI between the control group and birds receiving 5% fermented elephant grass (regardless of cultivar) suggests that moderate inclusion did not adversely affect diet palatability or voluntary intake. In contrast, higher inclusion levels (10%) tended to reduce growth efficiency, indicating that excessive fiber may exceed the digestive capacity of broilers, even when fermented.

Growth performance responses further highlighted the importance of cultivar selection. The higher ADG and FE of the CpGui5 treatment indicate that inclusion of this moderate inclusion (5%) of this variety can enhance growth performance, while higher inclusion levels (10%) may compromise nutrient utilization. This aligns with previous reports that maintaining a proper nutrient balance allows moderate fiber levels to improve broiler performance, but excessive fiber can impair digestibility and efficiency (Yadav and Jha, 2019). Therefore, elephant grass can be considered a sustainable forage component in poultry diets, provided its inclusion level is carefully optimized, and an appropriate variety is selected.

Muscle deposition and carcass yield are pivotal performance indicators in production efficiency and are sensitive to dietary fiber type, physicochemical properties, and inclusion rate. Excessive dietary fiber often delays feed transit and may reduce nutrient absorption, potentially diminishing carcass output (Shahin and Abd Elazem, 2005; Yadav and Jha, 2019). Notwithstanding this, our study found that most elephant grass incorporated treatments maintained key carcass parameters (especially dressed weight, half-bore, and breast muscle rate) within acceptable ranges, paralleling findings where moderate fiber inclusion did not impair geese carcass traits (Liu et al., 2024). However, the CpGui10 group exhibited slightly lower metrics, which suggests that exceeding a threshold inclusion level may compromise muscle accretion, potentially due to excess insoluble fiber challenging digestive capacity (Shahin and Abd Elazem, 2005; Mourão et al., 2008). Conversely, treatments with moderate fermented elephant grass inclusion preserved carcass yield, supporting the proposition that elephant grass, when judiciously included, can be a sustainable forage component without detrimental effects on meat quality. These findings are consistent with previous reports indicating that moderate inclusion of fibrous ingredients such as oat hulls or soybean hulls does not impair carcass characteristics, whereas excessive fiber can negatively affect meat yield (Yadav and Jha, 2019; Rybicka et al., 2024). Thus, fermented elephant grass can be incorporated into broiler diets without negative effects on carcass performance, provided inclusion levels remain within a physiologically tolerable range.

Blood biochemical parameters are hallmarks of physiological status, reflecting liver and kidney integrity, protein metabolism, and overall systemic health (Onasanya et al., 2015). In our study, the key serum markers including liver enzymes (ALT, AST, γ-GT, ALP), proteins (total protein, albumin, globulin), and renal indicators (BUN, creatinine, uric acid) of the chickens in the fermented elephant grass incorporated treatments were not different from the control treatment. This underscores the fact that fermented elephant grass-incorporated diet did not exert hepatotoxic or nephrotoxic effects. These findings are consistent with the work by Eghaghaa et al. (2023), who observed that broilers fed diets with up to 25% pre-treated ensiled elephant grass showed no adverse effects on ALT, AST, or ALP levels, indicating preservation of hepatic function. Furthermore, parallel studies on poultry incorporating moderate dietary fiber or alternative forages report stable blood biochemistry and maintained homeostatic balance (Yadav and Jha, 2019), supporting the interpretation that such inclusions can be physiologically benign when appropriately formulated. Taken together, these results support the safety of fermented elephant grass as a dietary component for broilers when appropriately formulated.

Gut microbial communities play a central role in nutrient digestion, immune modulation, and overall host health, making their diversity a critical indicator of intestinal ecosystem stability (Kogut and Arsenault, 2016). Measures of richness, such as Chao1 and ACE indices, provide insight into the number of species present, while diversity indices such as Shannon and Simpson account for both richness and evenness, reflecting the ecological balance of the community (Lozupone and Knight, 2008). In the present study, richness indices did not differ significantly among treatments, suggesting that fermented elephant grass inclusion did not affect the total number of bacterial taxa. However, the reduction in diversity in the CpPur5 group may be linked to dietary fiber type and concentration, which strongly influence gut microbial ecology. Different fiber sources provide distinct substrates for microbial fermentation, thereby shaping competitive interactions among bacterial populations (Jha and Mishra, 2021; Qiu et al., 2022). The reduction in diversity in CpPur5-fed chickens could also signal microbial specialization, where only specific taxa capable of utilizing elephant grass fiber proliferated, leading to reduced evenness. Such patterns have been observed in poultry and ruminants when diets rich in structural carbohydrates favored fiber-degrading bacteria, sometimes at the expense of overall community diversity (Qiu et al., 2022). While specialization may enhance the efficiency of fiber degradation, reduced microbial diversity can limit ecological resilience, potentially predisposing the host to dysbiosis under stress conditions (Sommer et al., 2017). Nevertheless, the maintenance of diversity in other treatment groups suggests that moderate incorporation levels of fermented elephant grass was tolerated by the gut microbiome, supporting microbial ecosystem balance and stability. This aligns with previous findings that gradual or moderate inclusion of unconventional fibrous feeds can enhance beneficial bacterial populations without compromising diversity (Pourabedin and Zhao, 2015; Yadav and Jha, 2019).

Beyond general diversity, the gastrointestinal tract of poultry harbors a complex microbial ecosystem in which a relatively stable core-microbiome coexists with more dynamic peripheral taxa. The core community provides fundamental metabolic, immunological, and ecological functions, ensuring homeostasis and resilience against perturbations such as dietary shifts (Kogut and Arsenault, 2016; Rychlik, 2020). In this study, a robust core microbiome comprising more than 90% of the OTUs was consistently maintained across all treatment groups, indicating that fermented elephant grass inclusion (regardless of cultivar or level) did not disrupt the foundational microbial scaffolding of the broiler gut. The persistence of this core community indicates that fermented elephant grass inclusion, irrespective of cultivar or inclusion level, did not disrupt the foundational microbial structure essential for gut stability and host health (Oakley et al., 2014; Waite and Taylor, 2014). The CpPur10 treatment exhibited the highest number of unique taxa, suggesting that higher inclusion levels may promote niche differentiation and the emergence of specialized microbial populations capable of utilizing complex structural carbohydrates. Such niche enrichment has been reported in poultry fed diets with elevated fiber content and may reflect adaptive microbial responses rather than adverse perturbations (Yadav and Jha, 2019).

At the compositional level, fermented elephant grass inclusion altered the relative abundance of several dominant bacterial genera without introducing entirely new taxa, indicating diet-driven restructuring rather than microbial replacement. Genera associated with fiber degradation and short-chain fatty acid production, including *Ruminococcaceae*_UCG-014 and unclassified_*Lachnospiraceae*, were enriched in specific treatments. These taxa are widely recognized for their roles in cellulose and hemicellulose breakdown and for producing metabolites such as acetate and butyrate that support intestinal epithelial integrity and immune regulation (Biddle et al., 2013). These metabolites not only provide a direct energy source for intestinal epithelial cells but also serve as important signaling molecules regulating inflammation, gut barrier integrity, and host metabolism (Koh et al., 2016). LEfSe analysis further demonstrated that dietary treatments selectively enriched functionally relevant taxa, such as *Megamonas* in CpGui5 and *Ruminococcaceae*_UCG-014 in CpGui10, while no discriminant taxa were identified in CpPur5, consistent with its reduced diversity indices.

Overall, the microbial results indicate that fermented elephant grass inclusion preserved a stable core microbiome while selectively modulating functionally important bacterial populations in a cultivar- and dose-dependent manner. Yadav and Jha (2019) demonstrated that fibrous ingredients promoted beneficial genera such as *Ruminococcaceae* and *Lachnospiraceae*, enhancing nutrient fermentation and improving intestinal health in poultry. Similarly, Qiu et al. (2022) emphasized that dietary fiber acts as a selective substrate, fostering the proliferation of cellulolytic bacteria and enhancing functional metabolic outputs of the gut microbiota. Other studies in poultry (Stanley et al., 2015; Rychlik, 2020) confirm that high-fiber diets support a more functionally diverse and resilient microbial ecosystem, which contributes to pathogen resistance and improved growth performance. These previous reports support that moderate inclusion of unconventional fibrous ingredients can support microbial functional capacity without compromising community stability. Therefore, the present study demonstrates that fermented elephant grass can be strategically incorporated into broiler diets to support growth performance, carcass quality, physiological health, and gut microbial homeostasis, provided that cultivar characteristics and inclusion levels are carefully optimized.

## 5 Conclusion

Incorporation of fermented elephant grass into broiler diets influenced growth performance and gut microbial composition in a cultivar- and inclusion level–dependent manner without adverse physiological effects. Chickens fed the Guiminyin cultivar at 5% inclusion exhibited growth performance and feed efficiency comparable to the control diet, whereas higher inclusion levels, particularly 10%, were associated with reduced growth and carcass indices, indicating a threshold beyond which fiber inclusion may impair nutrient utilization. Blood biochemical parameters related to liver and kidney function remained unchanged across treatments, confirming physiological safety. Gut microbiota analysis showed preserved microbial richness and a stable core microbiome across all diets, demonstrating resilience of the intestinal ecosystem to dietary intervention. However, microbial community evenness was reduced in the CpPur5 treatment. LEfSe analysis revealed treatment-specific enrichment of functionally relevant genera, including *Megamonas* in CpGui5 and *Ruminococcaceae_UCG-014* and unclassified *Lachnospiraceae* in CpGui10, while no discriminant taxa were identified in CpPur5. Overall, moderate inclusion of fermented elephant grass, particularly Guiminyin at 5%, supports productive performance while maintaining gut microbial stability.

## Data Availability

The raw data supporting the conclusions of this article will be made available by the authors, without undue reservation.
